# *Srebf2* Locus Overexpression Reduces Body Weight, Total Cholesterol and Glucose Levels in Mice Fed with Two Different Diets

**DOI:** 10.3390/nu12103130

**Published:** 2020-10-14

**Authors:** Irene Andrés-Blasco, Sebastian Blesa, Ángela Vinué, Herminia González-Navarro, José Tomás Real, Sergio Martínez-Hervás, Julián Carretero, Antonio Ferrández-Izquierdo, Felipe Javier Chaves, Ana-Bárbara García-García

**Affiliations:** 1 Genomics and Diabetes Unit, Health Research Institute Clinic Hospital of Valencia-INCLIVA, Menendez Pelayo 4acc, 46010 Valencia, Spain; iranbla@gmail.com (I.A.-B.); sebastian.blesa@ext.uv.es (S.B.); 2 Health Research Institute Clinic Hospital of Valencia-INCLIVA, 46010 Valencia, Spain; m.angela.vinue@uv.es (Á.V.); Herminia.Gonzalez@uv.es (H.G.-N.); Antonio.ferrandez@uv.es (A.F.-I.); 3 CIBERDEM (Diabetes and Associated Metabolic Diseases), 28029 Madrid, Spain; a.barbara.garcia@ext.uv.es; 4 Endocrinology and Nutrition Service, Clinic Hospital of Valencia, 46010 Valencia, Spain; jose.t.real@uv.es (J.T.R.); Sergio.martinez@uv.es (S.M.-H.); 5 Department of Medicine, University of Valencia, 46010 Valencia, Spain; 6 Department of Physiology, University of Valencia, 46010 Valencia, Spain; julian.carretero@uv.es; 7 Pathology Service, Clinic Hospital of Valencia, 46010 Valencia, Spain; 8 Department of Pathology, University of Valencia, 46010 Valencia, Spain

**Keywords:** cholesterol, atherosclerosis, lipoproteins, sterol regulatory element-binding protein 2 (SREBP-2), carbohydrate metabolism, lipid metabolism, high-fat, high-sucrose diet, transgenic mice

## Abstract

Macronutrients represent risk factors for hyperlipidemia or diabetes. Lipid alterations and type 2 diabetes mellitus are global health problems. Overexpression of sterol regulatory element-binding factor (*Srebf2*) in transgenic animals is linked to elevated cholesterol levels and diabetes development. We investigated the impact of increased *Srebf2* locus expression and the effects of control and high-fat, high-sucrose (HFHS) diets on body weight, glucose and lipid metabolisms in transgenic mice (*S-mice*). Wild type (*WT*) and *S-mice* were fed with both diets for 16 weeks. Plasma glucose, insulin and lipids were assessed (*n* = 25). Immunostainings were performed in liver, pancreas and fat (N = 10). Expression of *Ldlr* and *Hmgcr* in liver was performed by RT-PCR (N = 8). Control diet: *S-mice* showed reduced weight, insulin, total and HDL cholesterol and triglycerides (TG). HFHS diet widened differences in weight, total and HDL cholesterol, insulin and HOMA index but increased TG in *S-mice*. In *S-mice,* adipocyte size was lower while HFHS diet produced lower increase, pancreatic β-cell mass was lower with both diets and *Srebf2*, *Ldlr* and *Hmgcr* mRNA levels were higher while HFHS diet produced a rise in *Srebf2* and *Hmgcr* levels. *Srebf2* complete gene overexpression seems to have beneficial effects on metabolic parameters and to protect against HFHS diet effects.

## 1. Introduction

Metabolic alterations, mainly lipid metabolism disorders (LMD) and type 2 diabetes mellitus (T2DM), constitute a major global health problem according to the World Health Organization (WHO). There has been a fast increment in their incidence due to unhealthy lifestyle habits (sedentariness, excessive caloric intake), population ageing and genetic and epigenetic factors [1]. The lifespan of patients with these diseases is shorter compared to the general population [2]. The intake of a high-fat diet can lead to diet-induced obesity and metabolic disorders in humans that can be induced in the same way in rodents.

In order to understand the effects of macronutrients in humans, it is important to consider their effects in mice (including the analysis in lean as well as obese ones) to clarify the pathogenesis of the metabolic disorders [3,4,5]. The sterol regulatory element-binding protein (SREBP) system is one of the main regulators of body and cellular metabolism [6].

SREBPs precursors are inserted into the endoplasmic reticulum (ER). They contain two membrane-spanning domains responsible for this insertion. This form is inactive and cannot undergo transcription. SCAP (or SREBP-cleavage-activating protein) interacts with the C-terminal SREBPs domain and as a result SREBPs move from the ER to the Golgi. This SCAP-SREBPs interaction takes place when cells are sterol-depleted. Due to the action of two Golgi proteases (site 1 and site 2 proteases, or S1P and S2P), the mature and transcriptionally active SREBPs forms are released. (Supplementary Figure S1) Mature SREBPs forms are translocated to the nucleus and join to the SREBPs target genes promoters [7]. SREBPs are transcription factors involved in cholesterol, lipid and glucose metabolism [7]. They also participate in adipocyte differentiation and insulin-dependent gene expression [6,8].

The SREBP system as a whole has been associated with different diseases such as dyslipidaemia, obesity, blood pressure, insulin resistance, diabetes mellitus, non-alcoholic fatty liver disease, nonalcoholic steatohepatitis, and chronic kidney disease [6,9,10,11,12,13,14].

SREBP system regulation is complex; recent studies have shown that SREBP-2 activity regulates SREBF1c and SREBF1a in the liver, implying that SREBP-2 could modulate the function of the whole system [15]. Studies using transgenic animals have shown that increased truncated SREBP-2, without a regulatory domain, can lead to alteration of insulin secretion, lower pancreatic β-cell (PBC) count, diabetes, reduced fat reserves, lower weight, increased total cholesterol in blood and tissues, and non-alcoholic hepatic steatosis [6]. In humans, some studies associate *SREBF2* variants with hypercholesterolemia [16], insulin resistance, DM and liver steatosis [17]. The *SREBF2* gene contains *miR-33a* gene which has been related to gene regulation in relation to cholesterol synthesis and efflux, insulin secretion and metabolism [18,19,20,21].

The present study has investigated the effect of increased *Srebf2* locus expression on glucose homeostasis and lipid metabolism in mice, and the effect of control and high-fat-high-sucrose diet (HFHS). To this end, we generated a transgenic mouse (*S-mouse*) with limited overexpression of the normal SREBP-2 protein by including extra copies of the *Srebf2* locus (which therefore includes *miR-33a*, which is located in intron 16 of *Srebf2*) controlled by its own promoter. Our *S-mouse* could prove a useful model to study the effect of treatments regulating *Srebf2* gene expression (including miR-33a) and/or SREBP-2 activity.

## 2. Materials and Methods

### 2.1. Mice and Diets

Animal care complied with institutional guidelines and following the 2010/63/EU European Parliament directive. All experiments were approved by the Animal Ethics Committee of Valencia University. Animals were transfected by microinjection with a pStart-K vector containing the *Srebf2* mouse gene (pStart-K-mSrebf2) from BAC MGSCv37 C57BL/6J (vector from mouse chromosome 15 including *Srebf2* gene and 10 upstream and 5 downstream Kb (Cyagen, Santa Clara, CA, USA). Therefore, transgenic SREBP-2 mice *(S-mice*) used in this study carried extra copies of *Srebf2* gene, including the 5′and 3′ indicated regions. A scheme of the cloning strategy and vector used can be seen in Supplementary Figure S2. Transgenic mice were obtained after backcrosses into C57BL/6J background. Wild type (*WT)* C57BL/6J mice (Charles River Laboratories, Wilmington, MA, USA) were used as a control group. The introduction of the extra copy of the *Srebf2* gene was verified by quantitative PCR (data not shown) in both groups. Both groups were identified by genotyping to detect or not presence of the vector regions (one primer hybridizes in the part of the vector that is inserted and other in a close regions of the inserted mouse genetic region: *Srebf2*-1: ATTGCTAGGCTCCCATTCCAA and vector: TGAAGTCAGCCCCATACGAT) and by the analysis of the extra *Srebf2* copy by EOSAL-CNV [22], data not shown). Mice had access to food and water *ad libitum* and were exposed to a 12-h light–dark cycle. Mice were maintained on a control diet (Teklad Global 14% Protein Rodent Maintenance Diet, Envigo, Indianapolis, IN, USA) and at 8 weeks of age, male and female *S-mice* and *WT* mice were placed on a HFHS diet for 16 weeks (60% fat 24% sucrose. Ssniff, Spezialdiäten, Soest, Germany). The diets compositions are detailed in Supplementary Table S1. Other control groups (male and female *S-mice* and *WT* mice) were bred and kept on a control diet for 24 weeks in the same animal facility.

### 2.2. Food Intake Experiment

For evaluation of food intake experiments mice were separated into individual cages at least 2 days prior to experiments. The animals were separated according to sex and genotype. For determination of daily food intake mice were fed a known amount of control diet and the remaining food was measured after 48 h. The experiment was repeated three times with the same animals.

### 2.3. Metabolic Measurements

Measurement of levels of plasma triglycerides (TG), total cholesterol (TC) and non-esterified fatty acids (NEFAs) was performed in overnight-fasted mice with standard enzymatic procedures using, respectively, LabAssay™ Triglyceride (reference 290–63701), LabAssay™ Cholesterol (reference 290–63701) (FUJIFILM Wako Chemicals, Neuss, Germany), and Free Fatty Acid Quantitation Kit (reference MAK044-1KT, Merck KGaA, Darmstadt, Germany).

HDL-cholesterol (HDL-C) was determined after dextran sulphate/MgCl_2_ precipitation of the apolipoprotein B-containing lipoproteins [23] (Dextran sulfate sodium salt, reference 42867-5G Merck KGaA) using the LabAssay™ Cholesterol (reference 290–63701) (FUJIFILM Wako Chemicals)

The glucose tolerance test (GTT) was performed as follows: after fasting overnight, an intraperitoneal injection of glucose solution (2 g/Kg of body weight, BW) was given to mice (reference 49163, Merck KGaA). Analysis of plasma glucose and insulin was performed at different times with an Ascensia Elite glucometer (Bayer AG, Leverkusen, Germany) and an ELISA ultrasensitive anti-mouse insulin, respectively (reference 90080, Crystal Chem, Zaandam, The Netherlands) [24,25].

For the insulin tolerance test (ITT), mice fasted for 4 h. They were given an intraperitoneal insulin injection (0.5 U/Kg of BW (ACTRAPID, EAN: 8470007755029, NovoNordisk, Copenhagen, Denmark), and plasma glucose levels were measured as explained above.

To analyze insulin resistance (IR), HOMA-IR index was calculated applying the formula “fasting plasma glucose (mmol/L) × fasting plasma insulin (µIU/mL)/22.5”.

Determination of liver TG content was performed after tissue digestion and saponification in ethanolic potassium hydroxide followed and enzymatic measurement of glycerol content (Free Glycerol Reagent, reference F6428, Merck KGaA) [26].

### 2.4. Enzyme-Linked Immuno Sorbent Assay (ELISA)

Insulin levels were determined in isolated plasma from ethylenediamine tetraacetic acid (EDTA) disodium salt dihydrate blood (reference 324503, Merck KGaA, Darmstadt, Germany) from mice using an Ultrasensitive Mouse Insulin ELISA kit (reference 90080, Crystal Chem). The kit was used following the manufacturer’s instructions.

### 2.5. Liver, Pancreas and Fat Immunostainings

For immunohistopathological analysis, liver, pancreas and fat were sectioned from mice sacrificed by cervical dislocation after perfusion with PBS, and then fixed with 4% paraformaldehyde/PBS for 4 h and paraffin embedded. β-cell mass was calculated by two ways: area of pancreatic islets relative to total pancreatic area (%), and pancreatic islet number relative to pancreatic area. Identification of pancreatic islet was undergone by insulin immunostaining in 3–4 slides per mouse, separated by 125 µm.

The immunohistochemistry protocol consisted of incubation with primary antibody (mouse monoclonal anti-insulin 1/500 dilution, reference I2018, Merck KGaA) followed by biotinylated anti-mouse secondary antibody (1/300 dilution, reference sc-2039, Santa Cruz Biotecnology Inc., Dallas, TX, USA), streptavidin-HRP 1/2 dilution (reference TS-060-HR, Thermo Fisher Scientific Inc., Waltham, MA, USA) and DAB substrate (reference SK4100, Vector Laboratories, Burlingame, CA, USA). Slides were counterstained with haematoxylin and mounted with EUKITT (reference A10500, Deltalab, Barcelona, Spain). Images were captured with a digital stereo microscope with micro imaging LEICA DMD108 (Leica Biosystems, Wetzlar, Germany) and analyzed by computer-assisted morphometry with Image J (1.47v, NIH, available at https://imagej.nih.gov/ij/download.html). Lipid droplet content in liver and adipocyte content in fat was performed in haematoxylin-eosin stained section.

### 2.6. Adipocyte Quantification in Adipose Tissue: Size and Number

Adipocyte quantification in fat was performed in haematoxylin stained sections and analyzed by computer-assisted morphometry with Image J (1.47v, NIH, available at https://imagej.nih.gov/ij/download.html). Images were transformed from RGB to HSV color space with MRI adipocyte tools. To allow the application of standard morphological operations the image was then binarized. Adipocytes were counted, and absolute pixel area of each object was calculated and converted to μm^2^ [27]. The number and size of adipocytes were calculated and added to give values per animal.

### 2.7. RNA Extraction and Reverse Transcription from Tissue Samples

Total RNA from mouse liver was obtained using the Maxwell^®^ 16 miRNA Tissue Kit (reference AS1470, Promega, Madison, WI, USA). This kit allows the extraction of total RNA with enriched miRNA. The extraction kit was used according to the manufacturer’s instructions including the elution volumes quoted. RNA concentration and purity were measured using the NanoDrop ND-1000 spectrophotometer (Thermo Fisher Scientific Inc.) using 2 μL of RNA. RNA purity was evaluated using absorbance 260/absorbance 280 ratio: Ratios between 1.8 and 2.0 were assumed to be pure. All RNA samples were kept at −80 °C. To obtain cDNA, 500 ng of RNA per sample was reverse transcribed with Ready-To-Go You-Prime First-Strand Beads kit (reference 27926401, Cytiva, Marlborough, MA, USA). cDNA was diluted 1:10 in nuclease-free water and were stored at −20 °C.

### 2.8. mRNA Quantification by Real-Time Quantitative PCR

2 µL of diluted cDNA were used to perform the real-time quantification PCR using a 480 II real-time PCR system (Roche, Basel, Switzerland). The Kapa Sybr Fast qPCR master mix (reference KK4601 Roche) was used and all reactions were run in triplicate following manufacturer’s protocol, including blank/negative controls without cDNA. Data were analyzed with the apparatus software (version 1.5, LightCycler^®^ 480 Software, reference 04994884001, Roche, Switzerland), determining the threshold cycle (Ct) and the average of each sample. Concentrations of mRNA from two housekeeping genes, β-*actin* and *β-2-microglobulin*, were used to normalize gene expression and the product specificity was checked by melting curve analysis. Results were analyzed with the provided LightCycler^®^ 480 Software (version 1.5) (reference 04994884001, Roche). The 2−ΔΔCt2−ΔΔCt comparative method for relative quantification was used to calculate differences in expression levels for each target gene among samples. The primers designed with the primer express programme were as follows (Forward: Fw; Reverse: Rv): murine *actin-β:* Fw 5′-ACCAGTTCGCCATGGATGAC-3′ and Rv 5′-CAATGGGGTACTTCAGGGTCAG-3′; murine *Hmgcr* Fw 5′-CGTAACCCAAAGGGTCA AGATG-3′ Rv 5′-CAGACCCAAGGAAACCTTAGCC-3′; murine *Ldlr:* Fw 5′-AGTGTGATGGCCC CAACAAG-3′ Rv 5′-CACTCGTTGGTCTTGCACTCC-3′; and murine *Srebf2*: Fw 5′-TCCTTCACTT AACCATGTGATCC-3′ Rv 5′-ATGGTAGGTCTCACCCAGGAG-3′.

### 2.9. Statistical Analysis

Data are presented as the mean ± SEM. Differences were evaluated by one-way ANOVA analysis, and were considered statistically significant when *p ≤* 0.05 (GraphPad Prism v.5 software, GraphPad Software, San Diego, CA, USA). Outliers identified by Grubbs’ test were not considered for quantification (GraphPad Prism).

## 3. Results

### 3.1. Body Weight, Food Intake and Lipid Metabolism Characterisation of WT and S-mice Fed a High-Fat, High-Sucrose or Control Diet

**Figure 1:**
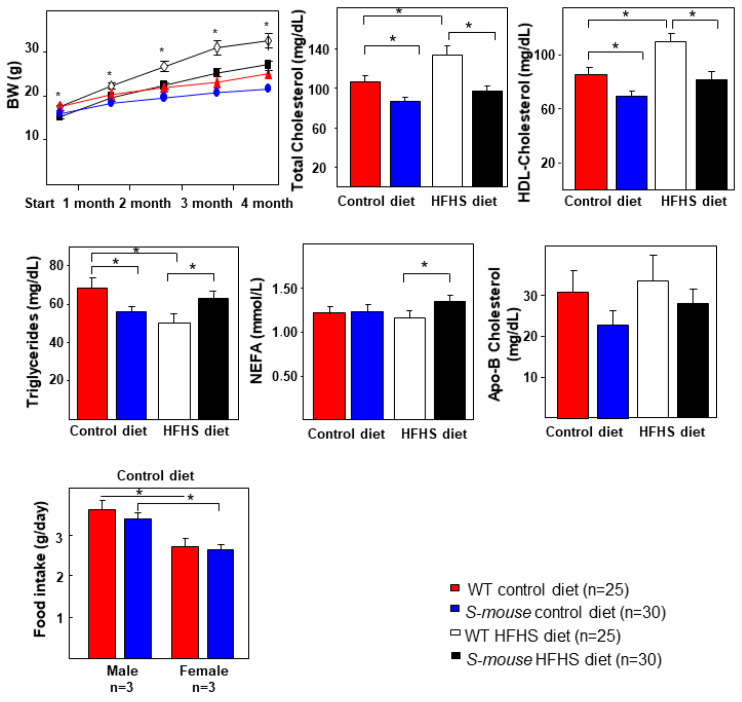
Plasmatic parameters in *WT* and *S-mice* fed a HFHS or control diet for 16 weeks. Evolution of body weight and total cholesterol, HDL-cholesterol, TG, NEFAs and apoB-cholesterol in fasted overnight levels in all mice groups. Data are presented as mean ± SEM. Statistical analysis was performed using one-way ANOVA. * *p* ≤ 0.05. HFHS high-fat, high-sucrose, BW body weight, NEFAS non-esterified fatty acid, HDL high-density lipoprotein, *WT* Wild type, TG triglycerides.

Eight-week-old male and female *WT* and *S-mice* were fed with a HFHS or control diets for 16 weeks and were characterized. Control diet group was composed by 12 *WT* males, 13 *WT* females, 15 *S-mouse* males and 15 *S-mouse* females. HFHS diet group was composed by 12 *WT* males, 13 *WT* females, 15 *S-mouse* males and 15 *S-mouse* females. Over the months studied body weight increased with age, but transgenic mice showed lower body weight than *WT* mice (Figure 1, top panel, *p* < 0.01). The HFHS diet increased the body weight in both genotypes and widened the difference between them.

Consistent with a role of SREBP-2 in lipid metabolism, lipid level analysis showed lower total cholesterol and HDL-cholesterol levels in *S-mice* fed a HFHS or control diet, although HFHS diet increased both total cholesterol and HDL-cholesterol in WT mice (Figure 1, *p* < 0.01). Triglycerides levels were lower in *S-mice* fed with control diet compared with WT mice but triglycerides were lower in *S-mice* under the control diet. HFHS diet increased triglycerides levels in *S-mice* while reducing triglycerides in *WT* mice. Finally, NEFAs is only higher in *S-mice* under HFHS diet when compared with *WT* mice (Figure 1, *p* < 0.05). No changes were observed in apolipoprotein B (apoB)-cholesterol (Figure 1). To determine the underlying mechanisms behind the low BW observed in *S-mice,* we performed food intake analysis on control diet fed animals. Surprisingly, animals under control diet had the same feeding. We did not find any statistical differences between genotypes. The food intakes ranged from 2.5 g/day/mouse to 3.5 g/day/mouse. Feeding behavior in individually housed mice during the early stages of control diet feeding demonstrates that *S-mice* consume the same food than *WT* animals, it should be noted that the *WT* and *S-mouse* females showed a lower food intake compared with males with same genotype. We monitored food consumption over one week under ad libitum conditions. No difference in food intake was observed between transgenic and *WT* mice but differences between sexes was significant (Figure 1, lower panel, *p* < 0.05). These differences in food intake between genders can have metabolic impact. In fact, if TG levels are separated by males and females under control diet (Supplementary Figure S3), there are differences in TG between *WT* males and *WT* females and they can be explained by this different food intake between males and females. However different food intake does not explain why TG levels in *S-mice* male and *S-mice* females under control diet are different.

### 3.2. Carbohydrate Metabolism Characterisation of WT and S-mice Fed a High-Fat, High-Sucrose or Control Diet

**Figure 2:**
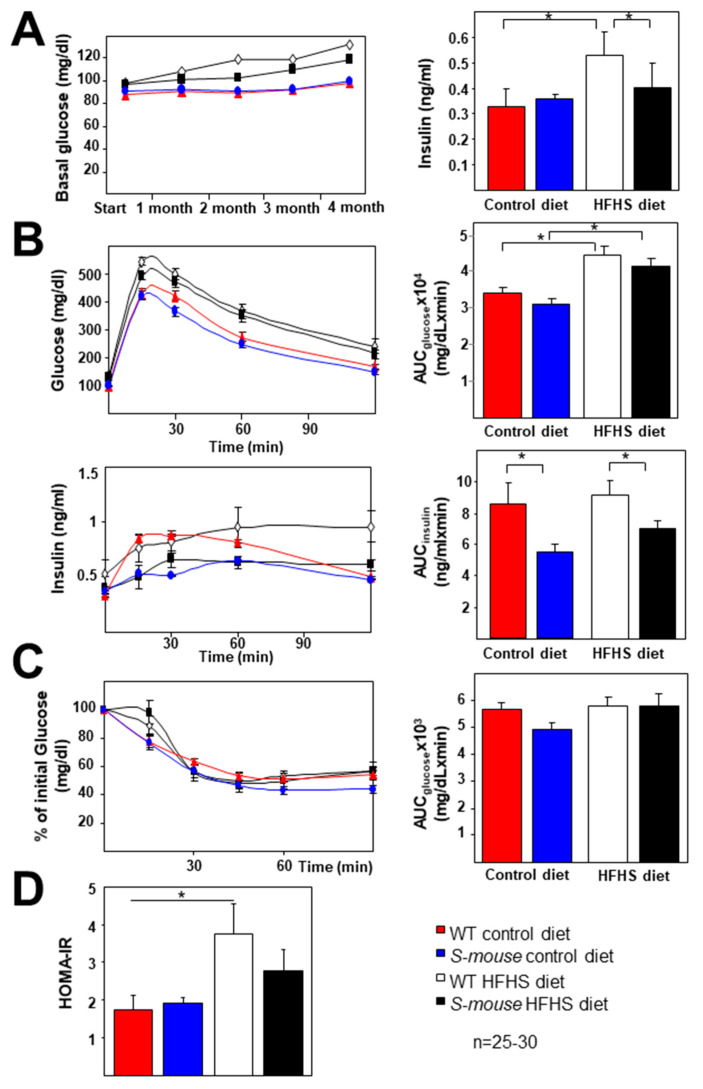
Glucose metabolism characterization under 16-week, high-fat, high-sucrose or control diet-fed *WT* and *S-mice*. (**A**) Evolution of fasting plasma glucose levels and insulin levels in all mice groups. (**B**) Plasmatic glucose levels during the GTT (top graph) and the area under the curve (AUC_glucose_) determined from the glucose measurements of the test (right panel) in all mice groups. Plasmatic glucose-stimulated insulin levels during the GTT (lower graph) and the AUC_insulin_ determined from the insulin measurements of the test (right panel). (**C**) Glucose levels (percentage relative to basal glucose levels) during ITT in 4 h-fasted mice. Statistical analysis was performed using one-way ANOVA. (**D**) HOMA-IR index in four groups of mice. * *p* ≤ 0.05. HFHS high-fat, high-sucrose, GTT glucose tolerance test, ITT insulin tolerance test, HOMA homeostasis model assessment.

Carbohydrate metabolism analysis showed no changes in fasting basal glucose and insulin levels between genotypes under control diet but HFHS diet increased glucose levels over time (Figure 2A). As expected, fasting basal glucose in *WT* mice fed a HFHS diet was increased compared with levels in *WT* mice fed a control diet and *S-mice* (Figure 2A, *p* < 0.05), and *S-mice* animals had higher levels on a HFHS diet than a control diet (Figure 2A, left panel, *p* < 0.05). Basal glucose was higher in *WT* mice under HFHS diet than levels in *S-mice*. Insulin levels were higher in *WT* mice fed with HFHS diet compared with *WT* mice fed a control diet and *S-mice* under HFHS diet (Figure 2A, right panel, *p* < 0.05). In this way, the HFHS diet effect in *WT* animals and in *S-mice* showed increased HOMA-IR index compared with *WT* and *S-mice* fed with a control diet (Figure 2D, *p* < 0.05). A trend was observed in the differences in HOMA-IR index between genotypes under HFHS diet (Figure 2D, *p* < 0.08).

Glucose tolerance test (GTT), measured as the area under the curve (glucose curve vs. time, AUC_glucose_) (Figure 2B), showed no differences between genotypes in mice fed a HFHS or control diet, although at 15 min the *S-mice* under HFHS diet had lower levels of glucose than *WT* mice on the same diet (*p* < 0.015). In this way, in *WT* animals fed a HFHS diet showed increased glucose levels during GTT compared with *WT* fed a control diet (Figure 2B, *p* < 0.05). The glucose-stimulated insulin release during the test, expressed as AUC_insulin_ (insulin curve vs. time) (Figure 2B, *p* < 0.05) showed lower levels in *S-mice* fed with either diet compared with *WT* mice. 

These data indicate that *S-mice* required lower insulin release for the same glucose levels and curves, although, in insulin sensitivity analysis by insulin tolerance test (ITT) AUC_glucose_ parameters showed no effect of *Srebf2-*overexpression in mice (Figure 2C). All these data may indicate that *S-mice* seem to be more protected against insulin resistance on a HFHS diet and overexpression of *Srebf2*.

### 3.3. Increased Dosage of Srebf2 Showed Low Pancreatic β-Cell Numbers in S-mice Fed a High-Fat, High-Sucrose and Control Diet

**Figure 3:**
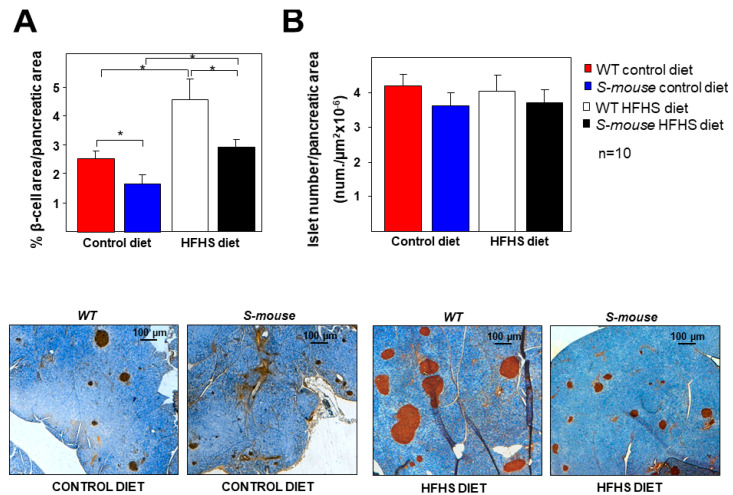
Pancreatic islet characterisation in *WT* and *S-mice* fed high-fat, high-sucrose or control diet for 16 weeks. (**A**) Quantification of β-cell area (in percentage relative to pancreatic area) and (**B**) relative islet number in the pancreas of all groups of mice identified by anti-insulin immunohistochemistry. Representative images of the immunohistochemistry are shown. Data are presented as mean ± SEM. Statistical analysis was performed using one-way ANOVA. * *p* ≤ 0.05. HFHS high-fat, high-sucrose.

To explore the causes of the differences in glucose-stimulated insulin secretion between *WT* and *S-mice*, pancreatic characterization was then performed. Analysis of pancreatic islets by insulin immunohistochemistry of cross-sections showed the relative area occupied by β-cells was significantly decreased in *S-mice* fed 16 weeks HFHS and control diet compared with their respective controls (Figure 3A, *p* < 0.05). The diet effect showed that *Srebf2*-overexpression in *S-mice* under HFHS diet increased β-cell levels compared to *S-mice* fed a control diet. Similarly, *WT* mice showed increased β-cell levels when fed HFHS diet as opposed to a control diet (Figure 3A, *p* < 0.05). Therefore, the HFHS diet produced an increase in β-cell levels in both animals, but the β-cell numbers were lower in *S-mice* (*p* < 0.05). No significant differences were observed in the islet number between the four groups of mice (Figure 3B).

These results indicate that the differences in insulin secretion were due to differences in β-cell mass and not to the number of islets.

### 3.4. S-mice Fed a High-Fat, High-Sucrose Diet Have Increased Hepatic Triglyceride Content Compared with S-mice Fed Control Diet

**Figure 4:**
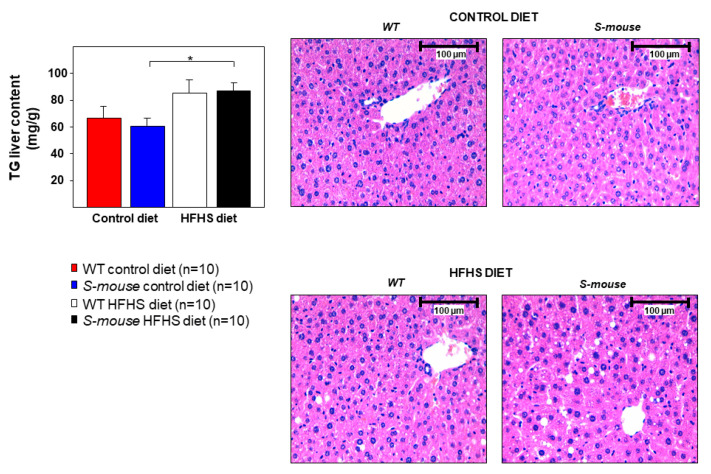
Liver characterisation in *WT* and *S-mice* fed high-fat, high-sucrose or control diet for 16 weeks. Analysis of triglyceride content in liver in all mice groups. Images of hematoxylin-eosin stained sections showing lipid droplets (20× magnification). Data are presented as mean ± SEM. Statistical analysis was performed using one-way ANOVA. * *p* ≤ 0.05. HFHS high-fat, high-sucrose, TG triglyceride.

Bearing in mind the relationship between non-alcoholic fatty liver disease (NAFLD), dyslipidemia, glucose metabolism derangement and blood triglycerides levels, hepatic analysis was performed. The result showed no differences between genotypes in hepatic triglyceride content, indicating that *Srebf2*-overexpression has no effect on fatty liver disease in mice fed with HFHS or control diet. However, the diet effect showed increased hepatic triglyceride content in mice fed a HFHS diet compared with mice fed a control diet (Figure 4). These results indicate that *Srebf2*-overexpression does not play a relevant role in increasing fatty liver disease in mice fed with a HFHS diet.

### 3.5. Adipose Tissue Characterization in WT and S-mice Fed a High-Fat, High-Sucrose or Control Diet

**Figure 5:**
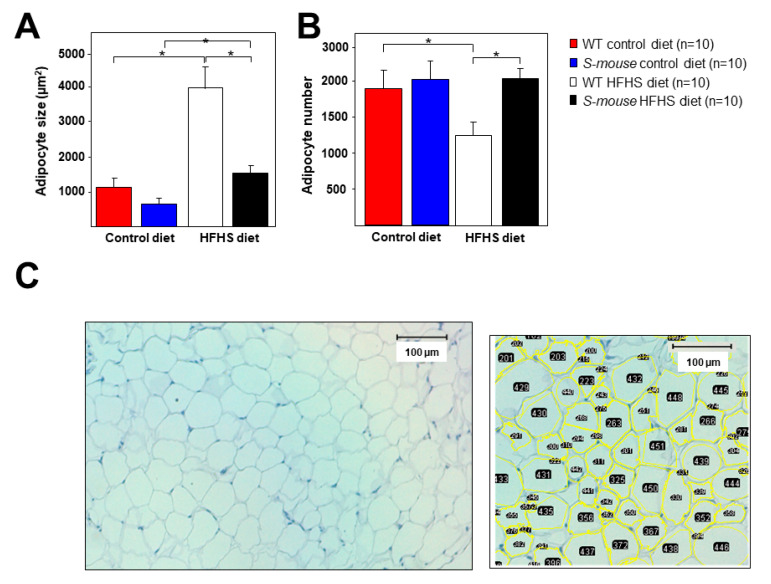
Adipose tissue characterization in *WT* and *S-mice* fed high-fat, high-sucrose or control diet for 16 weeks. (**A**) Quantification of adipocyte size and (**B**) adipocyte number in the adipose tissue for all groups of mice identified by hematoxylin-eosin staining. (**C**) Representative image quantified in A & B is shown. The ImageJ data acquisition detects the shape of the adipocyte and counts the number in the image. The same program shows the area of each adipocyte. Data are presented as mean ± SEM. Statistical analysis was performed using one-way ANOVA. * *p* ≤ 0.05. HFHS high-fat, high-sucrose.

Given the observed differences in body weight between different genotypes and the possible diet effect, adipose tissue was next analyzed. Adipocytes were characterized by haematoxylin-eosin stains in abdominal cross-sectional fat. These studies showed no differences in mice fed a control diet, although in *S-mice* tended to be smaller. Adipocyte size increased in *WT* and *S-mice* under a HFHS diet, although the increase in *WT* mice was much greater than in transgenic mice (Figure 5A, *p* < 0.01). Adipocyte number analysis showed reduced numbers in *WT* mice under HFHS diet when compared with *WT* mice under control diet and or *S-mice* under HFHS diet (Figure 5B, *p* < 0.01), probably due to their larger size. No differences were observed in mice fed a control diet (Figure 5B). Distribution diagram of adipocyte sizes in different diets and mouse genotypes can be watch in Supplementary Figure S4. These results indicate that *S-mice* have significant alterations in adipocytes metabolism or regulation, involving reduced capacity for body weight gain.

### 3.6. Increased Activation of Srebf2, Ldlr and Hmgcr in S-mice

**Figure 6:**
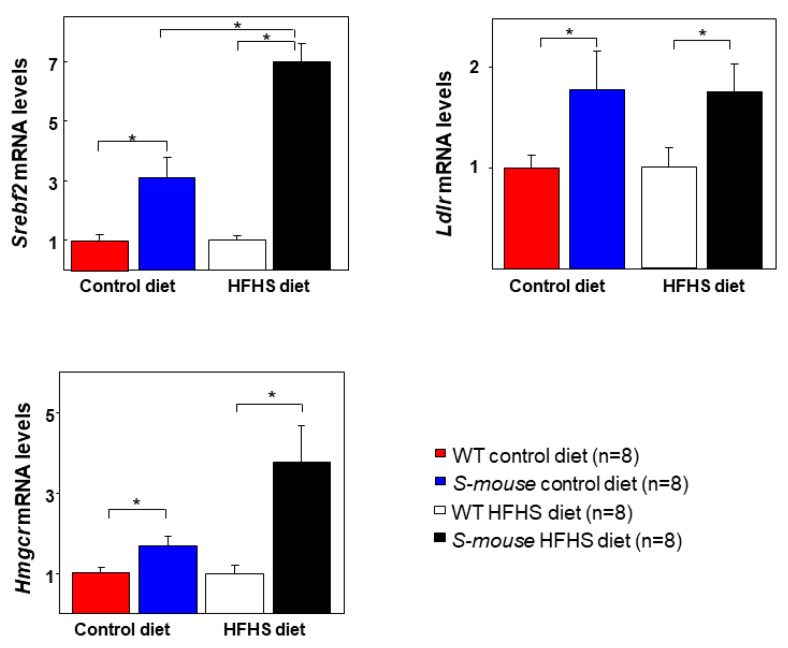
Expression analysis in liver in *WT* and *S-mice* fed high-fat, high-sucrose or control diet for 16 weeks. *Srebf2, Ldlr* and *Hmgcr* hepatic mRNA levels were normalized to *actin-β* expression. Relative expression shown was calculated using the 2^−(ΔΔCt)^ method. Statistical analysis was performed using one-way ANOVA. * *p* ≤ 0.05. HFHS high-fat, high-sucrose, *Srebf2* Sterol regulatory element-binding transcription factor 2, *Ldlr* Low-density lipoprotein receptor, *Hmgcr* 3-Hydroxy-3-methylglutaryl-CoA reductase.

To verify *Srebf2* overexpression and to understand these metabolic alterations and gene expression regulation by *Srebf2* we analyzed the mRNA levels of *Srebf2* and two genes regulated by *Srebf2* (*Ldlr* and *Hmgcr*). We found increased mRNA levels of *Srebf2*, *Ldlr* and *Hmgcr* in *S-mice*. These mice fed with HFHS showed increased *Srebf2* and *Hmgcr* mRNA levels (Figure 6, left panels, *p* < 0.05). In the same way, *S-mice* fed a control diet showed higher *Srebf2* levels than *WT* mice fed a control diet (Figure 6, left panel, *p* < 0.05). HFHS diet increased *Srebf2* mRNA levels in *S-mice* compared with mRNA levels in *S-mice* fed a control diet (Figure 6, left panel, *p* < 0.05). Similarly, analysis revealed that *Ldlr* mRNA levels were also higher in *S-mice* under both diets (Figure 6, right panel, *p* < 0.05) than in WT animals. Lastly, *Hmgcr* mRNA expression was significantly higher in *S-mice* fed a HFHS diet compared with those of *WT* mice fed a HFHS diet (Figure 6, low panel, *p* < 0.05). In the same way, *S-mice* under control diet showed enhanced *Hmgcr* levels compared with those for *WT* mice fed a control diet (Figure 6, low panel, *p* = 0.05). These results indicate that *Srebf2* overexpression increases the mRNA levels of *Srebf2*, *Ldlr* and *Hmgcr* in livers of *S-mice* fed with a HFHS and control diet and that *Srebf2* overexpression most likely increase the mRNA levels of other genes regulated by the SREBP-2 system.

## 4. Discussion

To understand the role of *Srebf2* in metabolism we generated *S-mice* which include extra copies of *Srebf2* mouse gene in upstream and downstream regions (approx. 10,000 bp and 5000 bp, respectively). The following differences between *S-mice* and other *Srebf2* transgenic mice were found: moderate overexpression of *Srebf2* gene regulated by its own promoter, expression in normal tissues and under own gene regulation, production of complete SREBP-2 protein (regulated by normal SREBP system) and the presence of miR-33a in the genetic sequence used. These mice can provide important information about different metabolic alterations related to body mass index (BMI), glucose and lipid metabolism and how they are regulated.

Although previous transgenic constructions had very high expression in one or two tissues of the active form of SREBP-2 (including NH2 acidic transactivation domain and bHLH-Zip DNA binding domain), they lack the COOH-terminal regulatory domain which produces constitutively active SREBP-2 [28,29,30,31]. These modifications result in absence of SREBP-2 regulation at transcriptional and posttranscriptional levels. Finally, most constructs are based on cDNA, therefore they lack miR-33A. This miRNA is encoded by intron 16 of *Srebf2* gene in mice and *SREBF2* gene in humans, with important roles in gene metabolism regulation modulating SREBP-2 activity [18,32,33]. Expression levels of mirR33a are correlated to the levels of Srebf2 gene expression in some cell types [19,32,33,34]. Studies have shown that *Srebf2* activation increases cholesterol levels in blood, tissues and cells [28,29,31]. Overexpression of *Srebf2* increases lipotoxicity in different kind of cells, including PBCs and hepatocytes, inducing DM and non-alcoholic steatohepatitis [29,35,36,37]. Moreover, very high overactivity of active SREBP-2 peptide in PBCs induces low weight, lower PBC mass, death and impaired insulin secretion in mice, which develop severe diabetes [29]. SREBP-2 overactivity in liver increases body weight, cholesterol and triglyceride synthesis levels and inhibits IRS expression in mice [28,38]. In rats, SREBP-2 overactivity in liver produces similar effects as in mice: reduced serum insulin levels and increased glucose levels [30]. Another study shows that mice with one *Srebf2* copy have reduced levels of glucose, postprandial reduced levels of insulin, reduced weight and triglycerides [39]. To our knowledge, although studies describe a notable point mutation affecting SREBP-2 functionality in mice, there is no data about glucose metabolism in these mice [40].

We demonstrated that an extra copy of *Srebf2* gene that can produce overexpression in cells where it is usually expressed has a significant effect on BW, T2DM and LMD parameters. Under a control diet, *S-mice* showed reduced BW, insulin secretion in GTT, and reduced levels of total and HDL cholesterol and TG. The HFHS diet produced or increased differences with *WT* mice in BW, total and HDL cholesterol and basal insulin and HOMA. This diet increased triglycerides and NEFAs plasmatic levels in *S-mice* compared to *WT* mice (Figure 1, Figure 2, Figure 3). Under HFHS diet *S-mice* gained body weight, but proportionally less than *WT* mice. Body weight increased by about 27% in *S-mice* while in *WT* the increase was about 32% (Figure 1, *p* < 0.001). These data are consistent with those found in mice overexpressing active SREBP-2 in PBCs caused low BW [29] and others [28] that found similar weight changes in male mice overexpressing active SREBP-2 in liver and fat. In this way, adipocyte size increased in both animals with a HFHS diet but in *S-mice* the increase is about 250% while in *WT* it is about 370% (Figure 5, *p* < 0.01). These results concur with reduced adipogenic activity showed in SREBP-2 transgenic SHR rats, although these rats had increased BW [30]. One of the mechanisms involved in adipocyte size can be the increased cholesterol levels in these cells due to *Srebf-2* overexpression which can limit their size [41]. The food intake experiment over a 7-days period was not altered in *S-mice*. No differences were found in short-term food intake between *WT* and transgenic mice but males showed more feeding than females (Figure 1, *p* < 0.05). Our study confirmed that overexpression of SREBP-2 in mice does not affect food intake. The study showed that eight-week-old *S-mice* on a control diet did not increase food intake, and further assessment of feeding behavior confirmed that *S-mice* maintained level of food intake than *WT* animals when fed the same diet. Our data suggest that the increment in food intake is not the primary factor for the obesity phenotype in *WT* mice. While this difference in food intake is the same, it does not appear to be enough to drive the weight gain and metabolic dysfunction. The weight gain could be correlated with the effect of the overexpression of SREBP-2 and with an increase of energy deposits in fat, decrease in voluntary activity, a drop in energy expenditure [42,43].

In both mice types a HFHS diet induced higher basal glucose levels and glucose intolerance compared with control diet. After 16 weeks on the HFHS diet, the *S-mice* had lower glucose and insulin levels than *WT* mice. The HOMA index showed no differences between genotypes, but under the HFHS diet HOMA-IR increased in all animals and tended to be high in *WT* animals compared with *S-mice* (Figure 2D). These data may indicate that moderate *Srebf-2* overexpression allows improved glucose metabolism. The number of pancreatic islets is similar in all animals, indicating that moderate *Srebf-2* overexpression allows their development, in contrast with mice with very high expression of active SREBP-2 in PBCs [29]. PBC mass is lower in *S-mice* than *WT* mice under control and HFHS diets. These facts could explain reduced insulin secretion and plasma levels in *S-mice* and indicate that *S-mice* require lower insulin levels and do not expand pancreatic islets to the same degree as *WT,* as happens in other mice or depending on diets [44,45,46,47].

Analysis of mRNA levels in the liver of *Srebf2*, *Ldlr* and *Hmgcr* (the last two regulated by SREBP-2 and involved in cholesterol uptake and synthesis) revealed increased levels in *S-mice* and a notable rise under HFHS diet in *Srebp2* and *Hmgcr*, while there was no change in *Ldlr* with a HFHS diet. Induced LDLR overexpression by *Srebf2* might explain the reduced total cholesterol blood levels in both diets.

We found marked differences between *S-mice* and other transgenic animals in many parameters connected with metabolic alterations and the role of SREBP-2 in them. Most of these changes are increased by HFHS diet. The effects can be partly due to transcriptional and posttranscriptional regulation, but could also owe to metabolic regulation by miR-33a, not analyzed in the current work, as could HDL levels [31,32]. All these data indicate a relevant effect of *Srebf2* overexpression on hepatocytes, adipocytes and PBCs, which is increased by HFHS diet. In general, *Srebp-2* overexpression seems to have beneficial effects on all the measured metabolic parameters except HDL levels. SREBP-2 is an interesting pharmacological target for treating different diseases including DM, metabolic syndrome, dyslipidaemia and cancer [48,49,50]. Our results pave the way for future research on the organ-specific action and mechanisms involved in SREBP-2 to facilitate its use as a drug target.

In summary, the present study demonstrates that a moderate *Srebf2*-overexpression produces reduced weight and modulates lipid and glucose metabolism compared with *WT* mice. In addition, a HFHS diet increases these differences, suggesting a role for *Srebf2* in diet induced effects in relation to these metabolic alterations. In general, complete *Srebf2* gene overexpression improves metabolic parameters related to cardiovascular risk and further studies are justified to assess the viability of therapeutic strategies based around modulation of SREBP-2 and miR-33a.

## Supplementary Materials

The following are available online at https://www.mdpi.com/2072-6643/12/10/3130/s1, Figure S1: SREBP activation, Figure S2: Transfection vector, Figure S3: Plasma TG levels in male and female mice, Figure S4: Adipocyte size distribution, Table S1: Diets composition.
