# Supplementary material for: Srebf2 Locus Overexpression Reduces Body Weight, Total Cholesterol and Glucose Levels in Mice Fed with Two Different Diets
Source: Nutrients. 2020 Oct 14;12(10):3130. doi: 10.3390/nu12103130 (PMC7602228; doi:10.3390/nu12103130)
Supplement: Supplementary file 1 [file nutrients-12-03130-s001.zip › supplementary figures and table/Supplementary Table 1.pdf]

Supplementary Table 1. Diets composition

**Standard Diet**	
Macronutrients	
Crude Protein	14.3 %
Fat (ether extract)	0,00
Carbohydrate available	0,48
Crude Fiber	4.1 %
Neutral Detergent Fiber	18.0 %
Ash	4.7 %
Energy Density	2.9 (12.1) kcal/g(kJ/g)
Calories from Protein	20 %
Calories from Fat	13 %
Calories Carbohydrate	67 %
Minerals	
Calcium	0.7 %
Phosphorus	0.6 %
Non-Phytate Phosphorus	0.3 %
Sodium	0.1 %
Potassium	0.6 %
Chloride	0.3 %
Magnesium	0.2 %
Zinc	70 mg/kg
Manganese	100 mg/kg
Copper	15 mg/kg
Iodine	6 mg/kg
Iron	175 mg/kg
Selenium	0.23 mg/kg
Vitamins	
Vitamin A	6.0 IU/g
Vitamin D3	0.6 IU/g
Vitamin E	120 IU/g
Vitamin K3 (menadione)	20 mg/kg
Vitamin B1 (thiamin)	12 mg/kg
Vitamin B2 (riboflavin)	6 mg/kg
Niacin (nicotinic acid)	54 mg/kg
Vitamin B6 (pyridoxine)	10 mg/kg
Pantothenic Acid	17 mg/kg
Vitamin B12	0.03 mg/kg
Biotin	0.26 mg/kg
Folate	2 mg/kg
Choline	1030 mg/kg
Amino Acids	
Aspartic Acid	0.9 %
Glutamic Acid	2.9 %
Alanine	0.9 %
Glycine	0.7 %
Threonine	0.5 %
Proline	1.2 %
Serine	0.7 %
Leucine	1.4 %
Isoleucine	0.6 %
Valine	0.7 %
Phenylalanine	0.7 %
Tyrosine	0.4 %
Methionine	0.3 %
Cystine	0.3 %

**HFHS Diet**	
Proximate Profile	
Protein	20.5 %
Fat	36.0 %
Fiber	0.0 %
Ash	3.5 %
Moisture	<10 %
Carbohydrate	36.2 %
Caloric Profile	
Protein	0.82 Kcal/gm
Fat	3.24 Kcal/gm
Carbohydrate	1.45 Kcal/gm
Amino Acids	
Alanine	5.3 gm/kg
Arginine	7.3 gm/kg
Aspartic Acid	12.8 gm/kg
Cystine	0.6 gm/kg
Glutamic Acid	40.6 gm/kg
Glycine	4.9 gm/kg
Histidine	5.5 gm/kg
Isoleucine	11.0 gm/kg
Leucine	16.6 gm/kg
Lysine	14.8 gm/kg
Methionine	7.1 gm/kg
Phenylalanine	8.9 gm/kg
Proline	20.5 gm/kg
Serine	11.4 gm/kg
Threonine	8.7 gm/kg
Tryptophan	2.2 gm/kg
Tyrosine	11.4 gm/kg
Valine	13.0 gm/kg
Carbohydrates	
Monosaccharides	1.0 gm/kg
Disaccharides	243 gm/kg
Polysaccharides	113 gm/kg
Fatty Acids	
C18:2 Linoleic	36.6 gm/kg
C18:3 Linolenic	3.6 gm/kg
Total Saturated	141 gm/kg
Total Monounsaturated	162 gm/kg
Total Polyunsaturated	40.2 gm/kg
Minerals	
Calcium	5.6 gm/kg
Chloride	0.86 gm/kg
Copper	3.6 gm/kg
Chromium	0.41 gm/kg
Fluoride	11.0 gm/kg
Iodine	0.31 gm/kg
Iron	40.8 gm/kg
Magnesium	0.49 gm/kg
Manganese	46.7 gm/kg
Phosphorus	5.8 gm/kg
Potassium	5.6 gm/kg
Selenium	0.21 gm/kg
Sodium	571 gm/kg

Lysine	0.7 %
Histidine	0.4 %
Arginine	0.8 %
Tryptophan	0.2 %
Fatty Acids	
C16:0 Palmitic	0.5 %
C18:0 Stearic	0.1 %
C18:1ω9 Oleic	0.7 %
C18:2ω6 Linoleic	2.0 %
C18:3ω3 Linolenic	0.1 %
Total Saturated	0.6 %
Total Monounsaturated	0.7 %
Total Polyunsaturated	2.1 %
Other	
Cholesterol	0 mg/kg

Sulfur	668 gm/kg
Zinc	21.6 gm/kg
Vitamins	
Choline	1148 gm/kg
Folic Acid	0.75 gm/kg
Niacin	15.0 gm/kg
Pantothenic Acid	5.5 gm/kg
Pyridoxine	4.1 gm/kg
Riboflavin	2.3 gm/kg
Thiamin	3.0 gm/kg
Vitamin A	3162 IU/kg
Vitamin B12	40 mcg/kg
Vitamin D2	1000 IU/kg
Vitamin E	25.7 IU/kg
Vitamin K3 (Menadione)	0.52 gm/kg
Ingredients	
Lard, Sucrose, Casein, Maltodextrin, Mineral Mix, Vitamin Mix, DL-Methionine, Choline	

Composition of standard and HFHS diets, given by commercial companies
