# Supplementary figures and images for: Srebf2 Locus Overexpression Reduces Body Weight, Total Cholesterol and Glucose Levels in Mice Fed with Two Different Diets
Source: Nutrients. 2020 Oct 14;12(10):3130. doi: 10.3390/nu12103130 (PMC7602228; doi:10.3390/nu12103130)

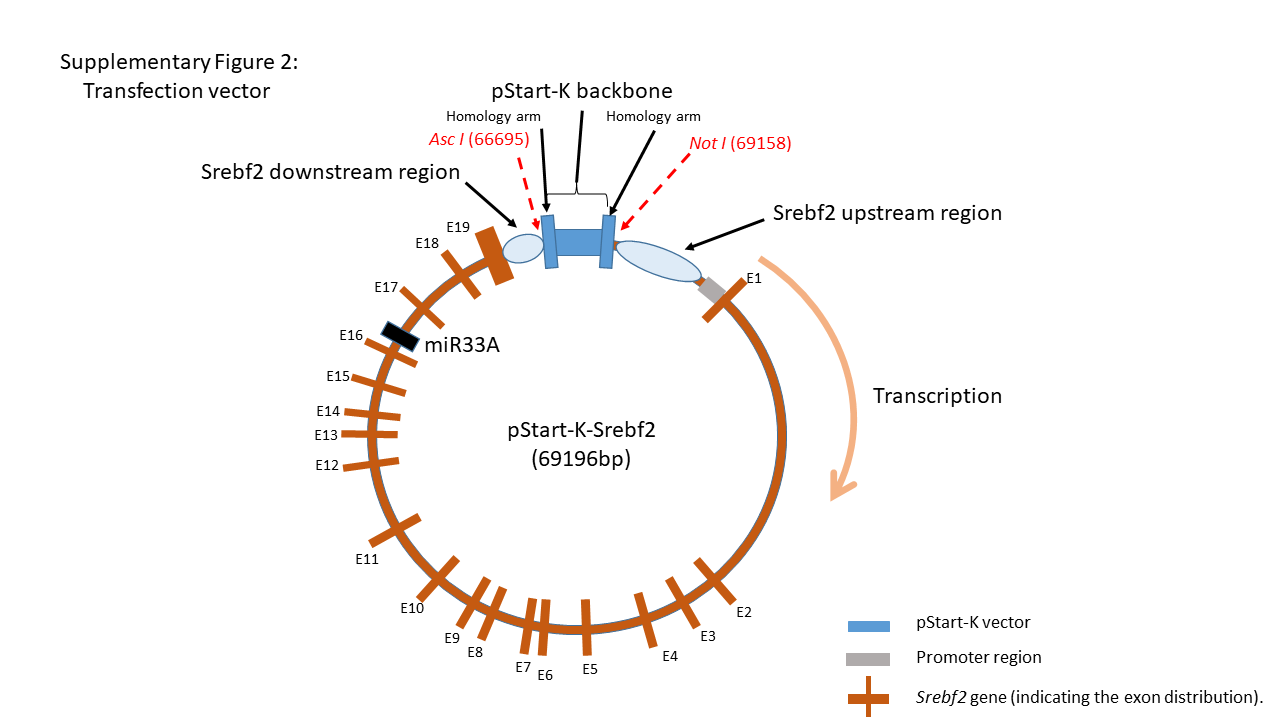

Supplement: Supplementary file 1 [file nutrients-12-03130-s001.zip › supplementary figures and table/Supplementary Figure 2.tif]

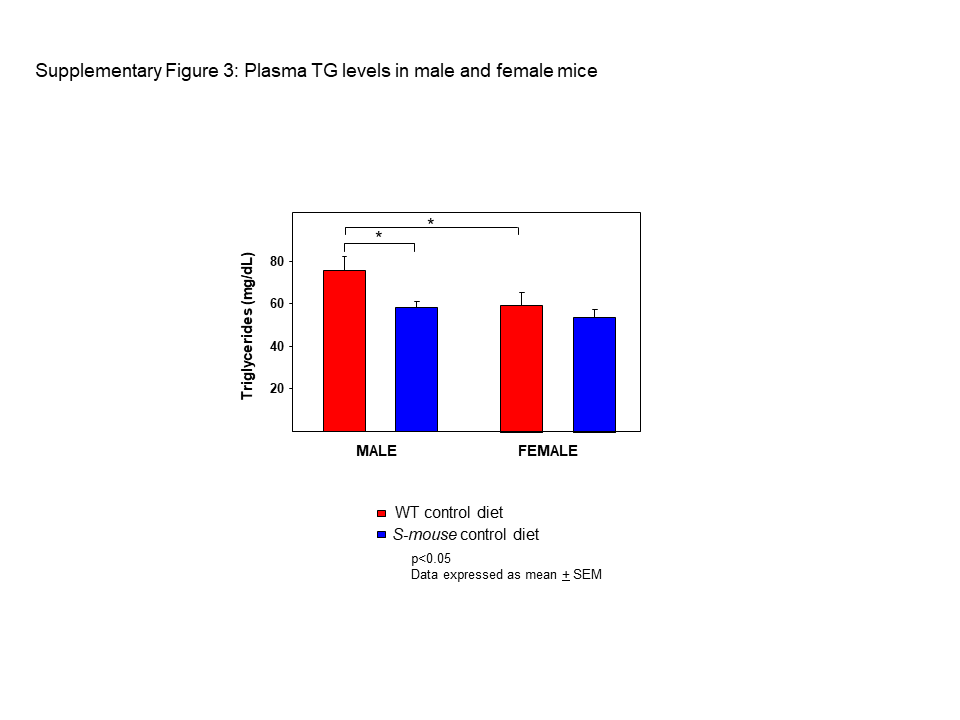

Supplement: Supplementary file 1 [file nutrients-12-03130-s001.zip › supplementary figures and table/Supplementary Figure 3.tif]

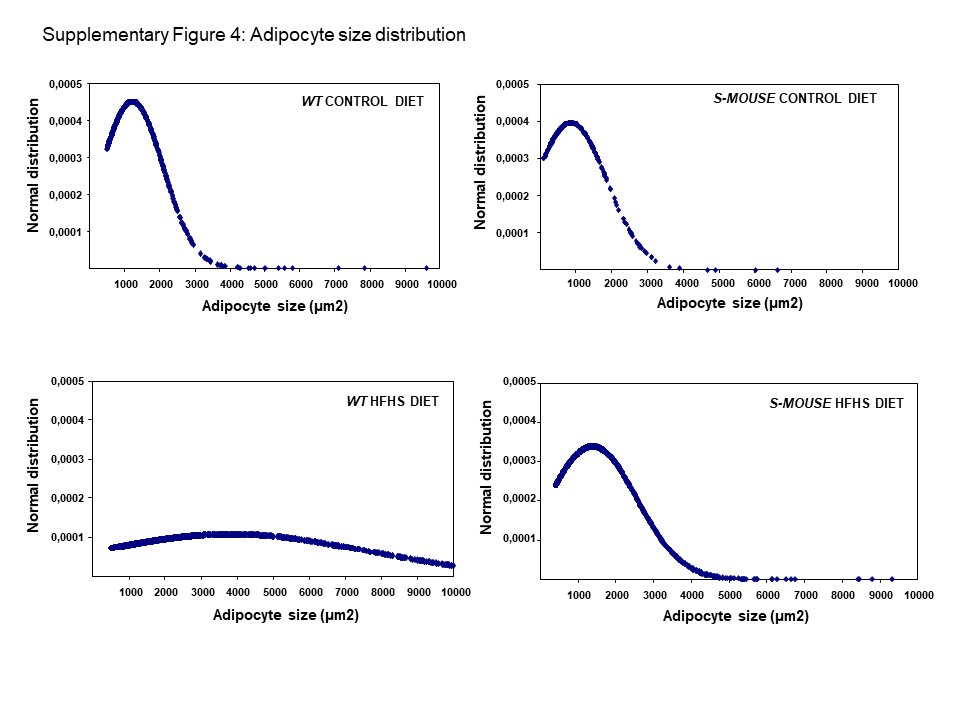

Supplement: Supplementary file 1 [file nutrients-12-03130-s001.zip › supplementary figures and table/Supplementary Figure 4.tif]

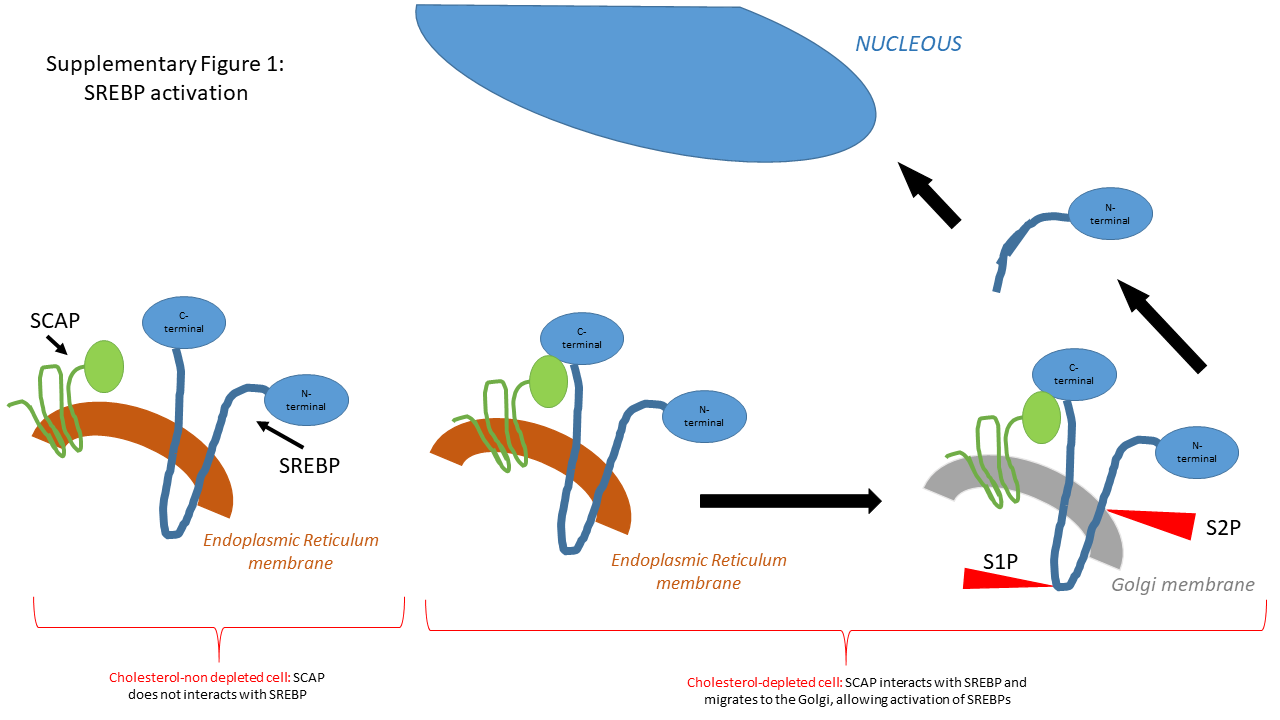

Supplement: Supplementary file 1 [file nutrients-12-03130-s001.zip › supplementary figures and table/Supplementary_figure-1.tif]
